# The combined application of hand hygiene and non-sterile gloves by nurses in a tertiary hospital: a multi methods study

**DOI:** 10.1186/s13756-024-01378-5

**Published:** 2024-02-28

**Authors:** Mireille Dekker, Rosa van Mansfeld, Marjon Borgert, Jolanda Maaskant, Frederique Paulus, Annamarike Seller, Irene P. Jongerden

**Affiliations:** 1 Department of Medical Microbiology and Infection Prevention, Amsterdam UMC, Vrije Universiteit Amsterdam, Boelelaan 1117, Amsterdam, The Netherlands; 2 Amsterdam UMC, Department of Intensive Care, University of Amsterdam, Meibergdreef 9, 1105 AZ Amsterdam, The Netherlands; 3 Amsterdam UMC, Department of Internal Medicine, University of Amsterdam, Meibergdreef 9, 1105 AZ Amsterdam, The Netherlands; 4 Department of Human Resources, Amsterdam University Medical Centers, University of Amsterdam, Meibergdreef 9, 1105 AZ Amsterdam, The Netherlands; 5 Amsterdam Research Center for Health Economics, University of Amsterdam, Amsterdam, The Netherlands; 6 Amsterdam UMC, Vrije Universiteit Amsterdam, Amsterdam Public Health research institute, Societal participation & Health, Amsterdam, the Netherlands; 7 Department of Public and Occupational Health, Amsterdam UMC, Vrije Universiteit Amsterdam, Amsterdam Public Health research institute, De Boelelaan 1117, Amsterdam, The Netherlands

**Keywords:** Infection Prevention, Nosocomial infections, Guideline Adherence, Cross-transmission, Glove-use, Hospitals, Behavior, Nursing.

## Abstract

**Background:**

The application of hand hygiene (HH) and the use of non-sterile gloves (NSG) in daily care is highly intertwined. We aimed (1) to assess the combined application of HH and NSG among nurses and (2) to explore determinants that influence their ability to combine both measures in their care.

**Methods:**

In a multi-methods study, we combined direct observations of care episodes with semi-structured interviews with nurses in two affiliated university hospitals. Topics were based on Flottorp’s checklist of determinants of practice.

**Results:**

In total, we observed 205 care episodes and interviewed 10 nurses. Observations revealed that the combination of NSG and HH was correctly applied in 19% of care episodes in which a single procedure was executed, and in 2% of care episodes in which multiple procedures were performed. From the interviews, we found determinants that influenced compliance, covered mainly by three out of seven of Flottorp’s checklist domains. Nurses indicated that their knowledge of protocols was limited to HH and protocols were hardly ever actively consulted; visual reminders within their workplace were used as sources of information. Nurses’ behavior was primarily influenced by their ability to operationalize this information and their ability to integrate both infection prevention measures into their care. The intention to apply and combine HH and NSG use was influenced by their risk assessment of cross-contamination, by the urge to self-protect and gut feeling. The feasibility to execute HH and NSG protocols is influenced by the urgency and the complexity of the care episode.

**Conclusions:**

The combined correct application with HH and NSG measures by nurses is low. Nurses are instructed in a fragmented way while in the day to day care HH and NSG use are highly intertwined. Operationalization and simplification of infection prevention protocols, in which instructions on both infection prevention measures are fused, should be considered. Strategies to improve practice should consider the power of habit and nurses urge to self-protect.

**Supplementary Information:**

The online version contains supplementary material available at 10.1186/s13756-024-01378-5.

## Background

Hand hygiene (HH) measures and the use of non-sterile gloves (NSG) in hospital care are highly intertwined. To adhere to these infection prevention measures, it is essential that health care workers (HCW) are familiar with the protocols that describe these measures, that HCW can properly recognize HH and NSG indications and that they understand how to operationalize these protocols and combine these measures during their day-to-day care.

HH is suggested to be the most important strategy to prevent that patients acquire nosocomial infections, the cornerstone of infection prevention. Apart from preconditions such as nail and skin care, the exposition of forearms and the removal of wrist and hand jewelry before starting to work, appropriate HH is defined as a combination of the correct indication and technique [1, 2]. NSG should be used in situations where a risk of direct contact with body fluids is anticipated, and during all patient care activities for patients with isolation precautions in place [1, 2]. The correct combination and proper application of HH and NSG can reduce the contamination of hands of HCW and hospital surfaces and consequently for microorganism to be transmitted from one patient to another. In other words, it will reduce the risk of cross-contamination and consequently reduce the risk of acquiring infections [1, 3].

Research shows that contamination of NSG during routine care is common [4, 5]. The most frequent source of cross-contamination is the tendency of HCW to perform multiple procedures within the care episode of a single patient or between patients without changing NSG and performing HH [6–8]. NSG are changed in only 28% of care episodes with indication [9]. The most common reason for HCW to wear NSG is their own judgement, which can be influenced by behavior of senior colleagues in their proximity and peer pressure [10–12]. The decision to wear NSG is commonly influenced by their (mis)perception of dirt, their feelings of disgust and the urge to self-protect, leading to the misuse and overuse of NSG [10–13]. It is, however, no common knowledge for HCW that NSG use is no absolute barrier to transmission. The effects of NSG in the prevention of nosocomial infection are overestimated, their misuse and overuse a waste of resources and a financial burden with a negative impact on the environment [9, 14–16]. HCW assess themselves better than observations reveal [9, 14]. In addition, many HCW still are not familiar with the worldwide accepted ‘Five Moments of Hand Hygiene’ [15].

Although the correct application of HH and NSG use is necessary to minimize the risk of cross contamination, to date, there is no study that reports on the overall combined compliance of these complementary infection prevention measures. In this study we focused on HH and NSG use by nurses in the hospital setting, as nurses can have a profound impact on the prevention of infection; they are involved in the provision of care in every area of the hospital [2]. We aimed (1) to assess the adherence to the combination of HH and NSG protocols of hospital nurses and (2) to explore the determinants that influenced their combined HH and NSG use.

## Methods

### Study design

In a multi-method study, we combined observations of NSG and HH practices in direct patient care with semi-structured interviews with nurses. We followed the guidelines for reporting observational studies, the Strengthening the Reporting of Observational Studies in Epidemiology (STROBE) Statement and the guidelines for reporting qualitative research studies, the Consolidated Criteria for Reporting Qualitative Research (COREQ) [17, 18].

### Ethical considerations

This study was assessed by the Medical Ethical Committee of Amsterdam UMC, location VUmc, who waived the need for ethical approval (number 2022.0418).

### Setting and participants

The study was performed in Amsterdam University Medical Centers, consisting of two affiliated university hospitals with inpatient wards and outpatient clinics divided over two locations. We observed the combined NSG use and HH practices of nurses in eight departments: two intensive care units and six normal care hospital units. These units represented a cross-section of the hospital, including both locations, surgical and non-surgical units, intensive and non-intensive care. In addition, we interviewed nurses from these departments. All nurses from the participating departments were eligible. Nurses were invited to participate by an email that was sent to the nurse managers of these departments. Eighteen nurses responded and received an information letter about the aim and procedure of the interview and the voluntary nature of the study. Nurses that returned a written consent were interviewed using a digital platform (https://zoom.us/).

### Data collection

#### Observations

To determine the compliance with the combined HH and NSG use, we developed an observation form, inspired by previous audit tools (Additional file 1) [11, 19]. This observation form was pilot tested and evaluated by two observers (MD, KA). In addition, our observations were performed unannounced and discrete but not covert; observations were performed mostly during morning hours, given their high density of care episodes. Observations were performed between February 2022 and April 2022 by one infection control practitioner (KA) and one researcher with a background in infection prevention (MD). Episodes of care were defined as all care provided to a patient by a nurse between entering and leaving the patient zone. Appropriate application was defined as compliant to the protocol on HH and NGS use. To distribute the observations across the departments, a minimum of ten care episodes in which NSG were worn per department were observed, and a minimum of 20 care episodes for intensive care units were observed.

#### Semi-structured interviews

To explore which determinants played a role in their practices, nurses were interviewed in a semi-structured way. The topic list was based on the checklist of determinants of practice (the TICD checklist) (Additional file 2) [20]. The TICD checklist was developed with the intention to provide a generic and comprehensive tool to design, execute, evaluate and report implementation research and quality improvement projects and has proven its usefulness in the context of infection prevention [20–22]. Two female researchers (MD, MB), familiar with the TICD checklist and trained in interview techniques, conducted the interviews between June 2022 and September 2022. After eight interviews no new information came forward. We planned two more interviews to check for data saturation. The interviews lasted between 16 and 53 min.

### Data analysis

#### Observations

Observations were summarized using descriptive statistics. We calculated overall compliance by the sum of all observed care episodes in which HH and NSG use were indicated and performed correctly, divided by the sum of all observed care episodes in which their combined use was indicated. We assessed the number of appropriate HH and NSG practices at the start, during and at the end of care episode i.e. before, during and after the indication for combined application, the differences in correct application between care episodes that consisted of a single and multiple procedures and the number of moments at risk for cross-contamination. This risk was calculated by the number of times that objects or surfaces were touched by nurses with potentially contaminated NSG within or outside the patient zone. The patient zone is defined as the patient, his/her immediate surroundings and surfaces frequently touched by HCW while caring for the patient conform the World Health Organization criteria [3].

#### Semi-structured interviews

Interviews were recorded and transcribed verbatim by one researcher (MB) and checked for accuracy by one researcher (MD). Two researchers (MD, IJ) independently read the transcripts several times to familiarize with the data and highlight relevant quotes. We used the Rigorous and Accelerated Data Reduction (RADaR) technique to analyse the interviews [23]. This technique consists of four consequent steps. In the first step, data formatting, one researcher (MD) constructed a data table and added all highlighted quotes from the interviews. In the second step, data coding, quotes were coded by using the determinants the TICD checklist or new codes when relevant. One interview was independently coded by four researchers (MD, RM, IJ, MB), two interviews were independently coded by three researchers (MD, RM, IJ), and differences in coding were resolved through discussion. As consensus was high, subsequent interviews were coded by two researchers (MD and RM or MD and IJ). After all interviews were coded, the coding process was discussed within the research team (MD, RM, IJ) with the aim to reach consensus and improve the quality of the coding. In the third step, data reduction, two researchers (MD, IJ) arranged the quotes based on their codes and prepared a reduced data table; quotes were removed by using ‘track changes’ when they did not contribute to answering the research question or were merely repetition of information. Data were further reduced in subsequent shorter tables. Codes were sorted according to the TICD determinants. Quotes that illustrated the determinants were selected. The reduced data table, codes and choices were discussed within the research team.

## Results

### The combined application of HH and NSG

In total, we observed 205 care episodes in all participating departments. In half of the observed care episodes NSG and HH were combined; in 40% of these episodes, their combined use was indicated. The proportion of care episodes in which the combined use was indicated was higher for intensive care units than for normal care units (Table 1).

**Table 1:** Distribution of the observed episodes of care

	Intensive care	Normal care	Overall
	*n (percentage) [95% confidence interval]*		
Total episodes of care	55	150	205
Episodes of care in which hand hygiene and non-sterile gloves were combined	36 (65.5) [52.3–76.6]	68 (45.3) [37.6–53.3]	104 (50.7) [43.9–57.5]
Episodes of care in which hand hygiene and non-sterile gloves were combined and indicated	30 (54.5) [41.5–67]	51 (34.0) [26.9–41.9]	81 (39.5) [33.1–46.3]

Intensive Care wards *n* = 2, Normal Care wards *n* = 6

#### The appropriate application of HH and NSG

The proportion of correct combined use of NSG and HH was higher directly after indication, i.e. after the care was provided (61.7%) as compared to before indication, at the start of the care episode (25.9%). Meaning that nurses were more prone to perform HH in combination with doffing their gloves after they provided care, than performing HH before donning NSG. In 2.0% of the care episodes that consisted of multiple procedures, the combined use of HH and NSG was indicated and performed in accordance with protocols. In care episodes in which a single procedure was executed, adequate combined use was 18.5% (Table 2).

**Table 2:** Number of appropriate non-sterile glove and hand hygiene practice episodes

	proportion (percentage) [95% confidence interval]
Hand hygiene performed and non-sterile gloves donned directly before indication	21/81^a^ (25.9) [17.6–36.4]
Non-sterile gloves changed and hand hygiene performed during care episode when indicated	4/51^b^ (7.8) [3.1–18.5]
Non-sterile gloves doffed and hand hygiene performed directly after indication	50/81 (61.7) [50.8–71.6]
Total single procedure care episodes performed in accordance with both protocols	15/81 (18.5) [11.6–28.3]
Total multiple procedure care episodes performed in accordance with both protocols	1/51 (2.0) [0.3–10.3]

^a^ Number of episodes of care in which the application of hand hygiene and non-sterile gloves was indicated

^b^ Number of episodes of care in which at least one non-sterile glove change was indicated

#### The risk of cross-contamination

The overall risk for cross contamination in care episodes where NSG were worn was higher during the provision of care (51.9%) compared to doffing NSG and performing HH after provision of care (33.3%). During the provision of care nurses most frequently touched objects or surfaces within the patient zone with their potentially contaminated NSG. After providing care, objects or surfaces outside the patient zone were most frequently touched (Table 3).

**Table 3:** Episodes of care associated with cross-contamination

	during care episode ^†^	after care episode ^†^
	*n (percentage) [95% confidence interval]*	
Total episodes of care associated with cross-contamination	42 (51.9) [41.1–62.4]	27 (33.3) [24.0-44.1]
Gloves after contact with body fluids, not removed or removed but hand hygiene not performed and		
- subsequently touch patient	21 (25.9) [17.6–36.4]	5 (6.2) [2.7–13.6]
- subsequently touch object or surface within the patient zone	30 (37) [27.3–47.9]	13 (16) [9.6–25.5]
- subsequently touch object or surface outside the patient zone	21 (25.9) [17.6–36.4]	16 (19.8) [12.5–29.7]
- subsequently touch another patient		1 (1.2) [0.2–6.7]

^†^*n* = 81 (Episodes of care in which the combined application of hand hygiene and non-sterile gloves was indicated)

### The determinants that influence nurses’ compliance

We interviewed ten nurses about their combined NGS and HH practices. Nurses from six departments participated. The majority of these nurses was female (9/10) and their median work experience was ten years (range 5–30 years). Six of the interviewees worked at a Normal Care department.

Influences on the combined use of HH and NSG are covered by six out of seven of the main determinants (in bold) of the TICD checklist, with an emphasis on two of these determinants: guideline factors and individual health professional factors. Within these main determinants, more than one subdeterminant or subcode (in italics) was identified. A third TICD main determinant, patient factors, influenced the two aforementioned determinants.

#### Guideline factors

Nurses described NGS use and HH as feasible, if they could be executed as separate protocols. When NSG and HH had to be combined, their application interfered with nurses’ workflow. The dampness of the hands after disinfection hindered the *compatibility* with the NSG protocol; it made donning of NSG more difficult.

Protocols were updated regularly during the COVID-19 pandemic, which also impacted the *clarity* and guidance on NSG use. Nurses mentioned they were confused and in doubt of the credibility of infection prevention policies after updates.

If protocols are constantly changing or protocols between organizations are different, you get agitated and you don’t really know what to do anymore. And then - this is my own feeling - people are less likely to follow the protocol, like ‘We have to provide care in a different way again? So what’s really the right manner? [Intensive Care nurse, interview 8]

Nurses emphasized that the *feasibility* of HH and NSG use and was hindered when the vital functions of the patient were at stake. In these cases nurses felt that guideline factors were not compatible with the patient needs, a determinant of the ***patient factors*** domain.

If I have two patients in one room. You provide care for one patient and the ventilator alarm goes off, the other patient needs endotracheal suctioning. So then you want to take off the gloves, disinfect, put on a new pair of gloves. Depending on how much of a hurry there is, gloves really do get changed, but disinfection will be skipped sometimes, because you are in some kind of hurry. ‘You treat first that kills first’. [Intensive Care nurse, interview 3]

We know how to do it and that is take off gloves, disinfect hands, wait and put on new gloves, but practice shows: the patient is lying on his side and the glove gets contaminated with a bit of stool. Now I can’t continue, those gloves have to come off. But we can’t leave that patient lying on his side for that long either, because then the alarms go off, he can’t breathe properly, so then I’ll put on new gloves, but won’t disinfect. [Intensive Care nurse, interview 3]

#### Individual health professional factors

Nurses referred to their HH and NSG use as automatic behavior, focusing on the actual care rather than on recognizing the risk for cross contamination. Some nurses underscored the interview as an intervention to awareness of their *professional behavior*.

Nurses *knowledge* of the protocols was most often limited to HH. Nurses could describe “My five moments of hand hygiene”, but did not know of the existence of a separate protocol for the use of NSG as a standard precaution. Nurses believed that NSG use was only described in isolation protocols, and stated that they had never actively consulted NSG protocols. The visual reminders within their workplace were mentioned as prompts.

Nurses recognized HH and NSG opportunities more easily directly before and after the provision of care. Nurse felt that they lacked the *skills* to operationalize HH and NSG change indications in between and during procedures within a single care episode. Nurses explicated that this operationalization is not taught. Relating to the domain of ***guideline factors***; nurse mentioned that protocols present HH and NSG use as single actions and ignore the complexity of combining these measures in everyday practice.

After the procedure, you doff the gloves and immediately proceed for instance to change an IV line. In between actions the hands are not disinfected. We often think ‘I start, I disinfect, I finish and I disinfect’. [Normal Care nurse, interview 5]

The *intention and motivation* of nurses to combine HH and NSG was influenced their risk assessment of both risk for the patient and risk for themselves. ***Patient factors*** that influenced nurses’ motivation were the perceived dirtiness of a patient, or procedure and the perceived risk for infections for patients.

When nurses perceived specific tasks or even the patient as dirty, they were more inclined to combine both measures. Nurses underlined that this assessment was shaped by prior experiences and triggered by visible contamination. Overall, nurses underlined that the intention to self-protect influenced their behavior.

One of my previous experiences, I had a small hole in my glove and my hand visibly dirty after doffing. So with that experience, I know that afterwards I’ll always have to disinfect my hands. [Normal Care nurse, interview 9]

Higher perceived risks for infections for patients facilitated HH and NSG practices. Nurses agreed that the need to perform HH and wear NSG was essential in situations where the risk for infections for patients were presumed as high.

In central lines […] you see that people change their gloves very consciously, in addition to handling alcohol swabs and plasters very consciously. When inserting materials, it is done very consciously, both by the doctors and nurses. [Intensive Care nurse, interview 3]

If nurses were able to plan and prepare their care in advance, they said they were better able to operationalize and embed their NGS and HH practices in the care to be provided. Some nurses explicated how they developed workarounds to avoid the removal of gloves between different tasks or procedures.

And what I also see, and it’s not allowed, is two gloves on each hand, a size L and a size M on top of each other. You start with size L, doff that pair, and continue with size M. This occurs especially in situations that involve feces, you always get feces on your gloves. Then you throw them away, but immediately you have your new gloves on so you can continue. [Intensive Care nurse, interview 3]

## Discusion

This study investigated the adherence to the combination of HH and NSG protocols of hospital nurses and the determinants that influences their combined application in daily practice. It became evident that that nurses struggle to consistently and correctly combine the application of protocols on HH and the use of NSG, especially in care episodes in which multiple procedures are combined. Nurses’ knowledge was most often limited to HH and protocols were hardly ever actively sought.

Our results suggest that nurses’ behavior is influenced by their limited familiarity with infection prevention protocols, their ability to operationalize infection prevention protocols and their ability to integrate of these measures into their care. The intention to apply and combine HH and NSG use is influenced by their risk assessment of cross-contamination, but most importantly by the urge to self-protect and often based on gut feeling. The feasibility to execute HH and NSG correctly is influenced by the urgency and the complexity of the care episode which influences nurses’ ability to organize and arrange their care in advance. When classified to Flottorp’s checklist of determinants of practice, these findings relate to all main determinants, except for ‘social, political and legal factors’ with an emphasis on ‘guideline factors’ and ‘individual health professional factors’, and ‘patient factors’ influencing both of these determinants [20].

In line with our findings, Lescure and colleagues found equivalent determinants of practice with nurses in long term care facilities [22]. Also, the urge to self-protect, fear for infection and disgust as strong influencers of NSG use, have been described in detail [12, 13].

Importantly, this study highlights that one of the factors that influences the behavior of nurses is their unawareness of the existence of infections prevention protocols regarding NSG use in routine care. Visual reminders within their workplace were used as sources of information. These reminders, as well as protocols, provide information per infection prevention topic. Thus, nurses are instructed in a fragmented way while in the day to day care HH and NSG use are highly intertwined. This might lead to the conclusion that multiple infection prevention protocols should be operationalized, combined and translated into department or workflow specific instructions; these instructions should interfuse both measures seamlessly. An exercise in which infection control link nurses can play a significant role [24]. When we supplement this idea with the recommendations to simplify protocols and remove conflicting instructions, it might be less challenging for nurses to combine infection prevention measures into their day to day care [14, 25]. These evidence based strategies might be particularly relevant for nurses with the desire to follow hospital policies [26].

In more complex or urgent care situations, nurses find it difficult to integrate infection prevention measures; a finding that is supported by our observations. In stressful or complex care episodes with multiple procedures, nurses tend to have their behavior influenced by more unconscious choices, prioritizing their focus on direct patient care. This is where automatic or habitual behavior comes into play [27]. Habitual behavioris widely known to be a significant part of the behavior of health care professionals [27, 28]. Although habitual behavior is difficult to change, theory suggests that breaking a habit and learning new habits will trigger sustainable behavior change [27, 29, 30]. Pothoff and colleagues found a positive relation between planning and clinical behavior mediated by habit [29, 30]. This planning can be promoted by two complementary processes. Action planning– the creation of an action plan– can help nurses to plan how and when and where they will perform HH and use and change NSG [31, 32]. In addition, coping planning– planning how to overcome barriers– will help nurses to have solutions ready when they encounter situations that might hinder the correct application of HH and NSG [32]. To promote the formation of these new habits, we should support nurses with implementation strategies that involve both action and coping planning. It will help nurses to form, practice and rely on new habits and, in parallel, have the head space to think and reason about the provision of direct, urgent and complex patient care.

### Strengths and limitations

To our knowledge, this is the first study to report the combined compliance of HH and NSG use. The use of Flottorp’s checklist and the involvement of an expert on its application increased the reliability and validity of our findings. We were able to identify and classify determinants of influence from a broad prospective. Triangulation of data from observations and interviews, underscored the complexity of the combined compliance with HH and NSG measures, especially when care is comprehensive.

Although our findings show substantial overlap with literature related to determinants of behavior regarding this subject, our study was only performed in two merging academic hospitals which limits its generalizability to other setting. The reliability of the observations may vary between the observers. We did try to limit inter observer variability by pilot testing and evaluating the audit form.

The nurses that participated in the interviews were contacted by their nurse managers. This sampling method may have affected the voluntary nature of their participation and increased the risk of including only highly motivated respondents. We were able to include nurses with a variety of years in work experience in intensive and normal care, distributed across the two locations of the hospital. It provided the possibility to explore the perceptions of nurses in various departments throughout our hospital. In addition, some nurses in our interviews were rather critical towards their own behavior, while others were more critical towards the measures itself. This makes this risk of bias less likely.

## Conclusions

To conclude, our study has demonstrated that compliance with combined use of NSG and HH by nurses is low. The results underscore the need to support them in their combined application of HH and NSG. Nurses are instructed in a fragmented way while in the day to day care HH and NSG use are highly intertwined.

Efforts to improve HH and NSG practices need to consider the simplification and operationalization of infection prevention protocols, in which instructions on both infection prevention measures are fused. Strategies to implement these instructions should consider the urge to self-protect and support the formation of new habits.

### Electronic supplementary material

Below is the link to the electronic supplementary material.

Supplementary Material 1

Supplementary Material 2

## Data Availability

The dataset generated and analysed during the current study and the coding tree (in Dutch) for the interviews are available from the corresponding author on reasonable request.

## Declarations

### Previous presentation

The findings in this manuscript were presented at the biannual ICPIC conference on September 13, 2023, in Geneva, Switzerland (Abstract P116).

## Abbreviations

HH: Hand hygiene
NGS: Non-sterile gloves
HCW: Health care workers
STROBE: Strengthening the Reporting of Observational Studies in Epidemiology Statement
COREQ: Guidelines for reporting qualitative research studies, the Consolidated Criteria for Reporting Qualitative Research.
TICD: The Checklist of Determinants of Practice
RADaR: Rigorous and Accelerated Data Reduction technique
