# Supplementary material for: The combined application of hand hygiene and non-sterile gloves by nurses in a tertiary hospital: a multi methods study
Source: Antimicrob Resist Infect Control. 2024 Feb 28;13:23. doi: 10.1186/s13756-024-01378-5 (PMC10903006; doi:10.1186/s13756-024-01378-5)
Supplement: Supplementary file 1 — Supplementary Material 1 [file 13756_2024_1378_MOESM1_ESM.docx]

**ADDITIONAL FILE 1.** Observation form for combined non-sterile glove and hand hygiene practices

| 1 | Were non-sterile gloves used during care episode? | - Yes | - No 🡪 End of audit |
| --- | --- | --- | --- |
| 2 | Were non-sterile gloves indicated? | - Risk for contact with body fluids, mucous membranes and/or non-intact skin - Handling of used, non-disinfected instruments - Isolation precautions - Indication not related to infection prevention | - No |
| 3 | Were non-sterile gloves donned immediately before the indication? | - Yes | - No |
| 4 | Was hand hygiene performed before donning of non-sterile gloves? | - Yes | - No |
| 5 | Was there an indication for changing the non-sterile gloves during the care episode? | - Yes, number of indications … | - No |
| 5a | Were non-sterile gloves changed? | - Yes   Sequence of non-sterile glove changes … | - No 🡪 risk for cross contamination   sequence of   - touching the patient … - touching items within the patient environment … - touching of items outside of the patient environment … - touching another patient … |
| 5b | Was hand hygiene performed between the donning and doffing of gloves? | - Yes Sequence of hand hygiene episodes .. | - No |
| 6 | Were non-sterile gloves doffed immediately after the indication for non-sterile glove use had ended? | - Yes | - No 🡪 risk for cross contamination   sequence of   - touching the patient … - touching items within the patient environment … - touching of items outside of the patient environment … - touching another patient … |
| 7 | Was hand hygiene performed after non-sterile glove removal? | - Yes | - No |
| 8 | Comments? |  | |

| Name auditor | Date | Department | Intensive care or normal care |
| --- | --- | --- | --- |
