# Supplementary material for: The combined application of hand hygiene and non-sterile gloves by nurses in a tertiary hospital: a multi methods study
Source: Antimicrob Resist Infect Control. 2024 Feb 28;13:23. doi: 10.1186/s13756-024-01378-5 (PMC10903006; doi:10.1186/s13756-024-01378-5)
Supplement: Supplementary file 2 — Supplementary Material 2 [file 13756_2024_1378_MOESM2_ESM.docx]

**ADDITIONAL FILE 2.**  Topic list based on the checklist of determinants of practice (TICD)

| 1. Can you describe how protocols are generally used in the department?  • How do you feel about these protocols  • What do you think of these protocols (clear, findable, applicable)  2. Can you describe your workflow regarding non-sterile glove use and hand hygiene when caring for patients?  • Can you provide examples  3. What are the main reasons for you to apply HH and wear NSHS in patient care?  • What facilitates  • What hinders  4. Can you describe moments in care when you do not adhere to the protocols?  • Can you describe the reasoning behind this  • Can you provide examples  5. Can you describe moments when you specifically do adhere to these protocols?  • Can you describe the reasoning behind this  • Can you provide examples  5. What would help you apply HH better and the same goes for wearing NSHS?  6. Are there any factors, circumstances, comments or other additions regarding applying hand hygiene, wearing and timely changing NSHS and   disinfecting hands that have not yet been discussed in this interview but that you would like to mention? |
| --- |
